# Environmental factors affecting the diversity of psychrophilic microbial community in the high altitude snow-fed lake Hemkund, India

**DOI:** 10.1016/j.crmicr.2022.100126

**Published:** 2022-03-17

**Authors:** Vidhu Gupta, Somashekar Chandran, Akash Deep, Rahul Kumar, Lalita Bisht

**Affiliations:** a Department of Environmental Sciences, H.N.B. Garhwal University (A Central University), Srinagar Garhwal 246174, Uttarakhand, India; b Department of Forensic Medicine and Toxicology, Adichunchanagiri Institute of Medical Sciences, Adichunchanagiri University, B.G. Nagar, Karnataka 571448, India; c Chair of Hydrobiology and Fishery, Institute of Agricultural and Environmental Sciences, Estonian University of Life Sciences (EMÜ), Fr. R. Kreutzwaldi 1, Tartu 51006, Estonia

**Keywords:** Actinomycetes, Bacteria, Fungi, High-altitude, Limnological parameters, Indian Himalayan region

## Abstract

The current examination incorporates the evaluation of limnological boundaries influencing the microbial diversity and its distribution in the Hemkund Lake, a high altitude aquatic body located at an elevation of 4,170 m a.s.l. in the Himalayan state Uttarakhand of India. Samples of water were collected for three continuous years (2018–2020) in three sampling attempts each year. Four water sampling sites were identified and studied across the lake during two years of the study periods. A total of nineteen physicochemical parameters of lake water were recorded. Few of the parameters were analyzed at the site whereas the leftover parameters were analyzed in the laboratory at the Department. The diversity of microorganisms was determined via morphological, biochemical, MALDI-TOF MS, and molecular approaches (16S and 18S rRNA sequencing). Environmental variables i.e., DO, BOD, total coliform, and TDS showed huge variation at site 2 among all the four water sampling sites. The water temperature of Hemkund Lake was observed from 4.9°C to 6.1°C whereas; dissolved oxygen was recorded from 6.0 to 8.2 mg.l^−1^. The α-diversity of microorganisms in the Hemkund Lake was found to be nineteen with ten bacterial strains, four actinomycetes strains, and five fungal strains. *Janthinobacterium lividum, Pseudomonas tolaasii, Pseudomonas rhodesiae, and Pseudomonas fluorescens* are a few important and key species that were found in the lake water*.* The present study on the diversity of psychrophilic microorganisms in the high altitude Lake Hemkund could be a great reference for further research activities on comparable viewpoints in different parts of the Himalaya. This baseline information can also help the administrative officials to take necessary steps for its conservation and management.

## Highlights

• Seasonal variation among the physicochemical attributes of Hemkund Lake.
• Exploration of psychrophilic microbial diversity of high-altitude snow-fed Lake Hemkund.
• This lake is located at an altitude of 4170 m a.s.l. and is also an important tributary of Lakshman Ganga.
• Study of important physicochemical factors affecting the microbial diversity at various sampling sites.
• Importance of phychrophilic microbial diversity to the society.

## Graphical abstract

**Figure:**
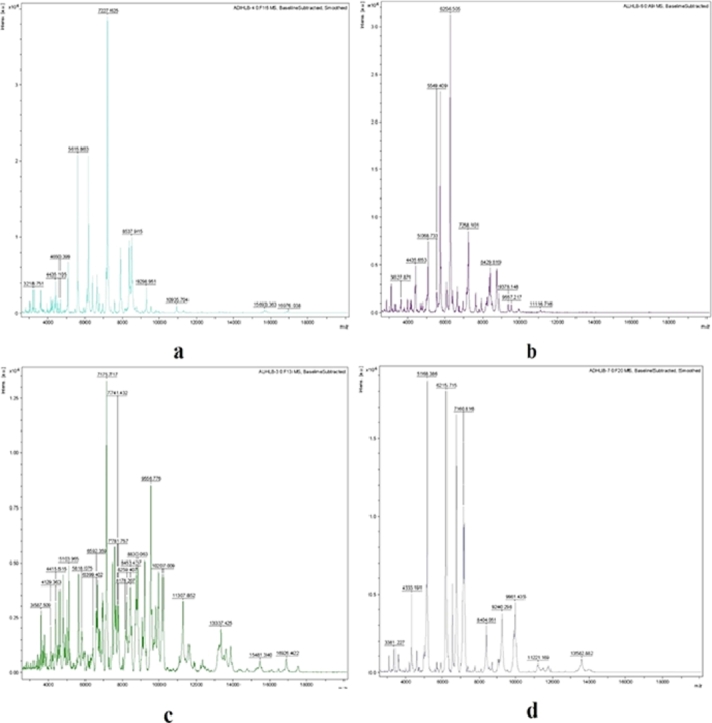

## 1 Introduction

Microbes are everywhere on the Earth. They can survive in all kinds of environmental conditions including physical and geochemical conditions (Rampelotto, 2010; Merino et al., 2019). Such microorganisms that are capable to survive, reproduce, and flourish under harsh environmental circumstances are very well known as extremophiles (Oarga 2009; Sharma et al., 2012). Depending on the physical environment (pH, temperature, salinity, pressure, radiation, etc) that they are required to survive, these extremophiles can be categorised into various categories. Based on temperature requirement for growth, these extremophiles can be categorised into three important groups including psychrophiles (+10°C to -10°C or less than this), mesophiles (10°C to 45°C), and thermophiles (more than 40°C. Each kind of extremophiles adopts their own cell plans and survival strategy and not changed since thousands of years. Psychrophilic microorganisms can survive and regenerate in extreme cold conditions within a temperature range of +10 to -10°C or even less than this i.e., polar regions, glaciers, glacier-fed lakes, snow-fed lakes.

High altitude snow-fed lakes are usually starting around 3000 m above m.s.l. (Pérez and Sommaruga, 2006) or the tree line. These lakes are usually having crystal clear water than the lakes located at lower and middle altitudes. These lakes are often surrounded by moraines, rocks, and boulders and are also known as Moraine-dammed lakes. High altitude lakes are primitive ecosystems because they are distant, difficult to reach and unaffected by human activity and characterized by low temperature, high UV radiation, unpredictable precipitation, low atmospheric temperature, low dissolved organic carbon, low nutrient content, and soil nutrient stress. Their geographical location exposes them to a variety of extreme environmental conditions. The harsh environmental conditions affect the hydrology and structure of the lake and may affect the microbial community and biochemical activities (Catalan et al. 2006; Liu et al., 2017; Rose et al., 2009; Wasserstrom et al., 2017). Microorganisms are the most abundant and important genetically diverse component of an aquatic ecosystem and play a key role in ecosystem processes and global biogeochemical cycles (Newton et al 2011; Liao et al. 2019). Therefore the lake ecosystems are ideal sites for studying the response of ecosystems to environmental changes and understanding microbial diversity and distribution in lakes can provide insight into biogeochemical processes and lake ecosystem functions (Dong et al 2010; Liu et al. 2015; Yang et al 2019). Absolute biomass and all fundamental biological system processes are dependent on them (Nazir et al. 2019). Microorganisms are significant parts of all environments; their omnipresence is mostly because of little size and simple dispersal, the capacity to develop and increase likewise under anaerobic conditions, their metabolic versatility and flexibility to use a wide scope of supplements. Microorganisms address the most extravagant stock of living molecular variety on the planet, establishing 60% of the microbial isolations could be important in farming, food and drugs industries; and this microbial diversity might be useful to decide the ecological status of the given ecosystem since they are also sensitive indicators of environmental health and quality.

Extremely cold environmental conditions favor the growth of microorganisms competent to survive under extreme cold commonly referred to as ‘Psychrophiles’ and cultured at low temperature. Psychrophiles have developed an intricate arrangement of morphological and physiological transformations for their endurance in outrageous cold ecological conditions (Pandit et al. 2014; De Maayer et al. 2014). Because of their potential use in molecular biology, dairy production, detergents, food additives, biosensors, ice creams, dietary supplements, it is important to understand the structure, diversity and function of microbial communities to fully understand the evolution and stability of life on Earth (Ley et al., 2005). A very less amount of scientific information on Hemkund Lake is available in the public domain. However, we can find some information on water quality and its physiography, but no information is available on microbial diversity and various factors affecting this diversity. The current study will definitely fill this research gap and will also provide key information on various environmental factors, microbial diversity, their importance, responsible factors affecting the water quality and microbial diversity of this important and sacred lake to the research community.

## 2 Materials and methods

### 2.1 The study area

**Fig. 1:**
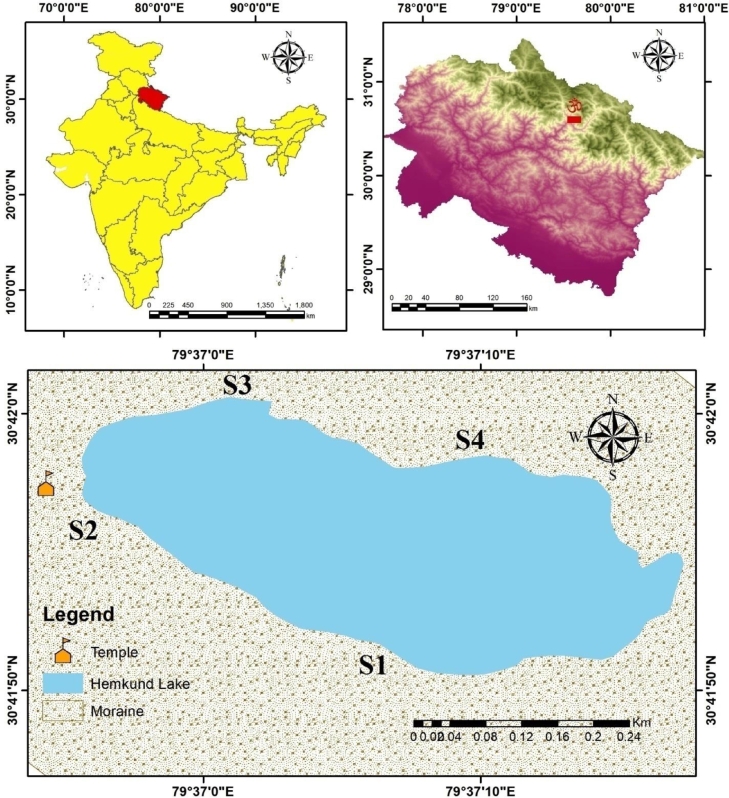
Location map of the sampling sites along the Hemkund Lake (4170 m above m.s.l.).

Hemkund Lake is placed at an altitude of 4170 m a.s.l. (latitude 30°41’55.17” N; longitude 79°37’05.79^”^ E) in the Uttarakhand state in India (Fig. 1). It is an oligotrophic lake surrounded by beautiful snow-capped peaks, moraines, and large boulders. The lake is fed by the ice and snow deposited on nearby mountain ranges especially the Hathi Parvat (Elephant Hill) and Sapt shring peaks and later forms a stream as its outflow known as Himganga. The lake stays under the frozen condition from September end to the furthest limit of May or sometimes June. Hemkund Lake is of irregular shape but somehow looks like a bowl that spread within a boundary/border of 1.48 km. The lake is around 0.10 km^2^ in area with 592 m length and 240 m width.

### 2.2 Water sampling

Four different sampling sites for sample collection (S1, S2, S3, and S4) were identified at the edges of the lake for sample collection depending on the lake's accessibility. Water samples of the lake were collected for three years (2018, 2019 and 2020) in three sampling attempts each year during June, August, and October as the lake remains under frozen condition for the remaining period. Water samples for physicochemical parameters were collected in sterilized polypropylene bottles whereas, water samples for microbial diversity were collected in vacuum flasks during the morning time and stored in a container filled with dry ice packs. Few of the important parameters that included pH, the temperature of air and water, free CO_2_, and dissolved oxygen (DO) were analyzed on-site. Later, the samples were shifted to the department for further analyses of the remaining parameters. Collected water samples were investigated at the laboratory for the remaining nineteen environmental parameters by following the standard methodology given in APHA (2012), Morello et al. (2003), Kumar et al., 2018 Kumar and Sharma (2021).

### 2.3 Physicochemical parameter analyses

A total of nineteen physicochemical parameters (air temperature, water temperature, free CO_2_, pH, DO, biological oxygen demand (BOD), turbidity, total dissolved solids (TDS), conductivity, alkalinity, hardness, sulphates, phosphates, nitrates, calcium, magnesium, chlorides, fecal coliform, and total coliform) were evaluated during the study period. Water and air temperatures were observed using a digital thermometer; nitrates, sulphates, and phosphates using a UV spectrophotometric method; free CO_2_, dissolved oxygen (Modified Winkler's method), hardness, calcium, and magnesium using titration methods; pH, conductivity, total dissolved solids, and turbidity using a multiparameter probe; sodium and potassium using the flame photometric method (APHA, 2012).

### 2.4 Coliform analyses

The coliform groups of bacteria are used as a water quality indicator that indicates the quality status of water whether it is fit for consumption or not. Both the widely accepted procedures for the detection of coliforms (multiple-tube fermentation method and membrane filter method) were used on MacConkey agar and broth media at 37°C. The multiple-tube fermentation (MTF) method involved three important tests to differentiate coliform and non-coliform groups of bacteria. These three tests were presumptive, confirmed, and complete. These tests were performed to culture the lactose fermenting gas-producing bacteria or coliform group of bacteria followed by their identification (APHA, 2012).

### 2.5 Microbial isolation and enumeration

Full strength (100%) and half-strength (50%) growth media were used to culture maximum microbial diversity (bacteria, fungi and actinomycetes). Nutrient agar media (HiMedia) was used to culture the bacterial colonies whereas, actinomycetes isolation agar media (HiMedia) was used to culture the colonies of actinomycetes. However, the Sabaroud Dextrose Agar media (HiMedia) enriched with ampicillin and streptomycin (50 mg.l^–1^ each) was used to isolate fungi. Ampicillin and streptomycin were added to avoid or to get rid of unwanted bacterial contamination. The pH of growth media and temperature of the incubator was adjusted according to the pH and temperature recorded at the sampling site. It was followed by the streaking of isolated microbial colonies on new plates of similar growth media using the streak-plate method to get the pure culture of each and every unique microbial colony (Clesceri et al. 1998, Kumar et al. 2020a).

### 2.6 Morphological and biochemical characteristics

Twenty-seven important morphological and biochemical traits were carefully examined during the study period to describe the detailed morphology of the microbial strains using naked eyes and a phase-contrast microscope (Nikon, Eclipse TS100). The morphological and biochemical assessments were performed to identify the lowest possible taxon of isolated microbial strains (Rohomania et al. 2015; Kumar et al. 2020b)

### 2.7 DNA extraction and gene amplification

After characterizing the microbial isolates using morphological and biochemical approaches, all the isolates were sent to the National Centre for Microbial Resources, Pune for further identification and confirmation through the Matrix-assisted laser desorption ionization-time of flight mass spectrometry (MALDI-TOF MS) method. Few of the microbial isolates that were not successfully identified through the MALDI-TOF MS method were again sent to the same facilitation centre for identification through the gene sequencing method. For such microbial isolates, “DNA was extracted using HiPurA Bacterial Genomic DNA Extraction Kit (HTBM008), and GSure Fungal DNA Extraction Kit (G45331). 16S rRNA gene was amplified by PCR using universal primers, forward primer 8F (AGAGTTTGATCCTGGCTCAG) and reverse primer 1492R (TACGGYTACCTTGTTACGACTT) (Takahashi et al. 2014) whereas, 18S rRNA gene amplification was performed by using specific universal ITS primers, forward 143 primer ITS 1F (TCCGTAGGTGAACCTGCGG) and ITS 4R (TCCTCCGCTTATTGATATGC)” (Pryce et al. 2003; Raja et al. 2017).

### 2.8 Identification of microbial isolates

#### 2.8.1 MALDI-TOF MS identification

MALDI-TOF MS is an emerging tool used for high-throughput and fast microbial identification. The principle behind microbial identification utilizing MALDI-TOF MS depends on the fact that each microorganism has a unique protein composition that gives its features and novel mass spectra. A mass spectrum of a strain is then compared with that of different strains present in the reference database (Fenselau and Demirev, 2001; Rahi et al. 2016). The facilitation centre provided the confirmed name of isolates along with their mass spectra.

#### 2.8.2 Molecular and *in silico* identification

Leftover strains that were not identified successfully through the MALDI-TOF MS method were sent again to the same facilitation center for gene sequencing (16S rRNA and 18S rRNA). The resulted consensus sequence of 16S rDNA was analyzed by Bio-Edit software *ver.*7.0.5.3 and submitted to NCBI. Then nucleotide sequences of newly identified microbes were collected from NCBI to find out the closest homologs by using Mega BLAST in order to characterize their genus. Divergence studies among 16S/18srRNA sequences of selected bacterial/fungal species were then subjected for Multiple Sequence Alignments (MSAs) using ClustalW *ver.* 1.6 (Thompson et al., 1994; Mohapatra et al. 2020) followed by construction of a phylogenetic tree using the maximum likelihood distance algorithm (Saitou and Nei, 1987) of MEGA 11 (Tamura *et al*., 2011; Mohapatra et al. 2016) program. The resultant tree topologies were evaluated by bootstrap analysis based on 1000 resampling.

### 2.9 Statistical treatment of data

PAleontological STatistics (PAST 4.07) (Hammer et al. 2001) software was used for the statistical treatment of physicochemical data of water samples.

## 3 Results

### 3.1 Water analyses

Samples of water were collected from snow-fed high altitude Lake Hemkund situated in the Uttarakhand state in India. The results of water analyses are mentioned below in Table 1. In the current study, the air temperature was recorded from 3.4 to 6.1°C whereas, the water temperature was recorded between 4.9 to 7.2°C. The pH values were reported from 7.06 to 7.72 exhibiting the nature of water as slightly alkaline. TDS was observed between 59 to 215 mg.l^–1^. The lowest (0.37 NTU) and highest (1.89 NTU) turbidity values were observed for lake samples. The lowest value of dissolved oxygen (6.0 mg.l^–1^) was reported at site 2 whereas, the highest value of dissolved oxygen (8.2 mg.l^–1^) was reported at site 1. Site 1 was reported for the lowest BOD value (0.2 mg.l^–1^) whereas site 2 and site 4 were reported for the highest BOD values (1.2 mg.l^–1^). The range of free CO_2_ that was assessed at the sampling site ranged between 2.2 to 11.0 mg.l^–1^. The value of total hardness was recorded from 4 to 24 mg.l^–1^ whereas, total alkalinity was reported to be 8 mg.l^–1^ at sites 2 and 4 and 50 mg.l^–1^ at site 2. Calcium and magnesium were ranged between 1.49 to 4.74 mg.l^–1^ and 0.61 to 1.63 mg.l^–1^. Conductivity was reported at the laboratory within a range of 27.93 µS/cm to 384.5 µS/cm. The highest value for sulphates was reported to be 0.171 mg.l^–1^. Values for nitrates and phosphates were reported between 0.100 to 0.574 mg.l^–1^ and 0.048 to 0.191 mg.l^–1^. Chloride values reported within 1.18 to 12.32 mg.l^–1^. Fecal coliform was totally absent in the water samples of Hemkund Lake during the research period. However, total coliform was ranged from 28 (CFU/100 ml) to 1321 (CFU/100 ml)

**Table 1:** Physiochemical analysis of water samples.

Parameters	Site 1			Site 2			Site 3			Site 4		
	Min	Max	X±SD	Min	Max	X±SD	Min	Max	X±SD	Min	Max	X±SD
**Air temperature (°C)**	**3.4**	5.7	4.60±0.80	4.1	**6.1**	5.12±0.73	3.6	5.7	4.69±0.73	3.6	5.6	4.73±0.69
**Water temperature (°C)**	5.1	6.9	6.02±0.69	5.5	**7.2**	6.38±0.60	**4.9**	6.8	5.98±0.68	**4.9**	6.7	5.98±0.62
**pH**	7.09	7.36	7.17±0.09	**7.06**	**7.72**	7.49±0.26	7.11	7.33	7.19±0.07	7.09	7.37	7.19±0.09
**TDS (mg.l^−1^)**	**59**	132	103.22±32.13	61	**215**	152.89±69.26	**59**	131	102.56±31.84	**59**	132	102.44±32.29
**Turbidity (N.T.U.)**	0.39	1.19	0.88±0.36	0.39	**1.89**	1.33±0.68	0.38	1.17	0.87±0.36	**0.37**	1.15	0.86±0.36
**DO (mg.l^−1^)**	6.2	**8.2**	7.33±0.77	**6**	8	6.69±0.89	6.8	8	7.27±0.53	6.8	8	7.27±0.47
**BOD (mg.l^−1^)**	**0.2**	1	0.67±0.26	0.4	**1.2**	0.89±0.38	0.4	1	0.69±0.23	0.4	**1.2**	0.73±0.30
**Free CO_2_ (mg.l^−1^)**	**2.2**	6.6	4.64±2.04	**2.2**	**11**	8.31±4.08	**2.2**	6.6	5.13±1.91	**2.2**	4.4	3.42±1.16
**Hardness (mg.l^−1^)**	**4**	12	8.67±3.32	8	**24**	18.67±6.24	6	12	8.89±2.67	**4**	10	7.56±2.40
**Total alkalinity (mg.l^−1^)**	18	37	28.22±8.11	14	**50**	37.33±15.62	**8**	36	24.89±12.77	**8**	32	21.78±10.41
**Calcium (mg.l^−1^)**	1.6	4.23	3.36±1.26	1.84	**4.74**	3.69±1.36	1.57	**4.74**	3.53±1.42	**1.49**	4.66	3.48±1.44
**Magnesium (mg.l^−1^)**	0.67	1.58	1.21±0.40	0.75	**1.63**	1.30±0.40	0.63	1.54	1.19±0.39	**0.61**	1.61	1.24±0.44
**Conductivity (μS/cm)**	31.84	259.7	175.63±107.70	41.21	**384.5**	259.79±161.20	30.27	261.8	177.41±110.14	**27.93**	231.1	159.01±97.97
**Sulphates (mg.l^−1^)**	**0**	0.073	0.05±0.03	**0**	**0.171**	0.11±0.08	**0**	0.079	0.05±0.03	**0**	0.065	0.04±0.03
**Nitrates (mg.l^−1^)**	**0.1**	0.419	0.31±0.15	**0.1**	**0.574**	0.40±0.22	0.103	0.427	0.31±0.14	0.103	0.379	0.27±0.12
**Phosphates (mg.l^−1^)**	0.06	0.109	0.09±0.02	0.067	**0.191**	0.14±0.05	0.06	0.113	0.09±0.02	**0.048**	0.098	0.07±0.02
**Chlorides (mg.l^−1^)**	1.42	11.36	7.42±4.59	1.42	**12.32**	8.29±4.65	1.36	11.36	7.33±4.55	**1.18**	10.94	6.84±4.30
**Fecal coliform (CFU/**100 ml**)**	0	0	0.00±0.00	0	0	0.00±0.00	0	0	0.00±0.00	0	0	0.00±0.00
**Total coliform (CFU/**100 ml**)**	48	384	266.00±148.69	76	**1321**	775.00±533.64	40	371	257.33±145.45	**28**	338	225.44±133.13

### 3.2 Statistical treatment of data

**Fig. 2:**
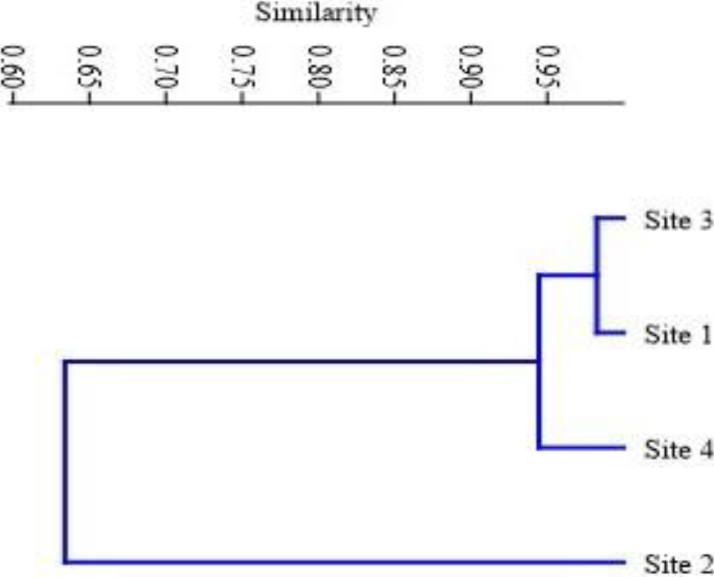
Bray-Curtis similarity index representing the similarity index between all four sampling sites.

The Bray-Curtis similarity index has been used to represent the similarity among four different sampling sites depending on the physicochemical attributes of water. Site 1 and site 3 showed the highest (0.98) whereas, site 4 showed a similarity index around 0.94 with that of site 1 and site 3. However, site 2 did not show any significant similarity with that of other sampling sites (Fig. 2).

### 3.3 Morphological and biochemical characterization

Twenty-seven morphological and biochemical tests were performed to characterize all the isolates of bacteria and actinomycetes. Shape, size, margin, elevation, color, cell shape, spore formation, flagella, and gram staining characterize the morphology of a bacteria whereas, catalase, citrate, urease, methyl-red, Voges-Proskauer, fructose, indole, raffinose, ribose, sorbitol, sucrose, xylose, trehalose, mannose, mannitol, lactose, and maltose were performed for biochemical characterization of a bacteria. Seven of the isolates (ADHLA-2, ADHLA-4, ADHLA-6, ADHLB-8, ADHLB-2, ADHLA-5, and ADHLA-7) were tested gram-positive whereas, the remaining isolates were tested gram-negative. The majority of the isolates were of circular shape having a size of 1–3 mm. Except for two isolates, all were tested negative for spore formation that means they were not spore forming isolates. The majority of isolates were peritrichous followed by atrichous and monotrichous. All the isolates were tested negative for raffinose and indole. All the isolates except one (ADHLB-8) were tested negative for methyl-red. Only three isolates (ADHLA-2, ADHLB-8 and ADHLA-5) were tested positive for the Voges-Proskauer test. Eight of the isolates (ADHLA-2, ADHLA-4, ADHLB-4, ADHLB-3, ADHLB-5, ADHLA-5, ADHLA-8, and ADHLB-7) were motile in nature and the remaining isolates were non-motile. The results of morphological and biochemical tests performed on the isolates of bacteria and actinomycetes are given in Table 2.

**Table 2:** Morphological and biochemical characteristics of bacterial isolates.

Characteristics	Bacterial Isolates											
	ADHLA-2	ADHLA-4	ADHLB-4	ADHLA-6	ADHLB-8	ADHLB-3	ADHLB-5	ADHLB-2	ADHLA-5	ADHLA-7	ADHLA-8	ADHLB-7
Shape	Round	Round	Circular	Circular	Circular	Circular	Circular	Circular	Round	Circular	Circular	Circular
Size	1–3 mm	4 mm	1–3 mm	2–4 mm	2–3 mm	2–3 mm	3–6 mm	1 mm	1–3 mm	4 mm	3 µm	2–5 mm
Margin	Entire	Undulate	Wavy	Rhizoid	Entire	Irregular	Regular	Entire	Entire	Entire	Irregular	Smooth
Elevation	Centrally raised	Semi-raised	Centrally raised	Raised	Convex	Raised	Convex	Convex	Centrally raised	Convex	Flat	Convex
Color	Sand yellow	White	Cream	Cream	Golden yellow	Cream	Hemolytic	Yellow	Cream	White	Opaque	Violet
Cell shape	Rod	Straight rods	Rod	Rod	Coccus	Rod	Rod	Ovoids to short rods	Rod	Coccus	Rod	Rod
Spore formation	+	+	-	-	-	-	-	-	+	-	-	-
Motility	Motile	Motile	Motile	Non-motile	Non-motile	Motile	Motile	Non-motile	Motile	Non-motile	Motile	Motile
Grams staining	Gram +ve	Gram +ve	Gram -ve	Gram +ve	Gram +ve	Gram -ve	Gram -ve	Gram +ve	Gram +ve	Gram +ve	Gram -ve	Gram -ve
Flagella	Peritrichous	Peritrichous	Peritrichous	Monotrichous	Atrichous	Peritrichous	Single Polar Flagellum	Atrichous	Peritrichous	Atrichous	Monotrichous	Single Polar Flagellum
Catalase	-	+	+	+	+	+	+	+	-	+	+	+
Citrate	+	-	+	+	+	+	+	-	+	+	+	+
Urease	-	+	-	-	+	-	-	-	-	-	-	-
Methyl Red (MR)	-	-	-	-	+	-	-	-	-	-	-	-
Voges Proskauer	+	-	-	-	+	-	-	-	+	-	-	-
Fructose	+	-	-	+	+	+	+	+	+	+	+	+
Indole Test	-	-	-	-	-	-	-	-	-	-	-	-
Raffinose	-	-	-	-	-	-	-	-	-	+	-	-
Ribose	-	+	-	-	-	+	+	-	-	-	+	+
Sorbitol	+	+	-	+	-	-	+	-	+		+	+
Sucrose	+	+	-	-	+	+	+	-	+	+	-	-
Xylose	-	-	-	-	-	+	+	-	-	-	+	+
Trehalose	-	+	-	-	+	+	+	-	-	-	-	-
Mannose	-	+	+	-	+	+	+	+	-	+	+	+
Mannitol	-	+	-	+	+	-	+	-	+	+	+	+
Lactose	-	+	+	-	+	-	-	-	-	+	-	-
Maltose	-	+	-	+	+	-	+	+	-	-	-	+

Abbreviations: +: positive; -: negative

### 3.4 Microbial diversity

**Fig. 3:**
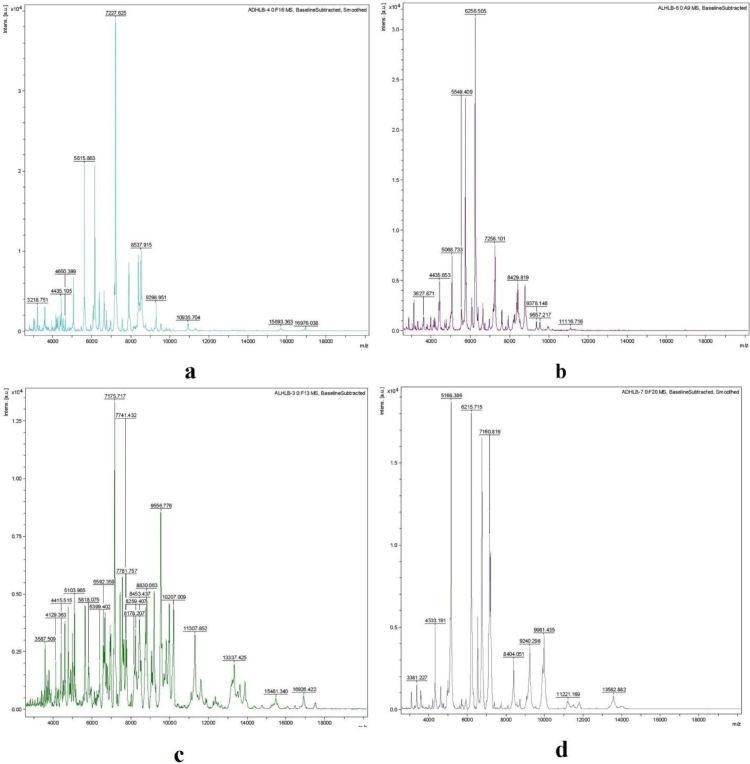
MALDI-TOF MS spectra of bacterial strains (a: *Pseudomonas rhodesiae*; b: *Pseudomonas tolaasii*; c: *Pseudomonas fluorescens*; d: *Janthinobacterium lividum*) indicating the protein profile (2–20 KDa).

A total of nineteen (10 bacteria; 4 actinomycetes; 5 fungi) microbial isolates were isolated from the water samples of Hemkund Lake (Table 3) that were carefully characterized for their morphology and biochemistry. A unique strain ID was assigned to all microbial isolates. Morphological and biochemical tests, MALDI-TOF MS and gene sequencing were made it possible to identify all the microbial strains up to their species level. After performing all tests we identified strain ADHLB-4 as *Ralstonia eutropha*, ADHLB-2 as *Neomicrococcus aestuarii*, ADHLA-8 as *Pseudomonas rhodesiae*, ADHLB-3 as *Pseudomonas fluorescens*, ADHLB-5 as *Pseudomonas extremorientalis*, ADHLB-7 as *Janthinobacterium lividum*, ADHLB-8 as *Staphylococcus aureus*, ADHLA-4 as *Bacillus licheniformis*, ADHLB-1 as *Paenibacillus glucanolyticus*, ADHLB-6 as *Pseudomonas tolaasii*, ADHLA-2 as *Streptomyces clavifer*, ADHLA-5 as *Streptomyces Rangoon*, ADHLA-6 as *Microbacterium schleiferi*, ADHLA-7 as *Arthrobacter polychromogenes*, ADHLF-1 as *Aspergillus cvjetkovicii*, ADHLF-2 as *Aspergillus sydowii*, ADHLF-7 as *Cladosporium fulvum*, ADHLF-3 as *Aspergillus jensenii*, and ADHLF-5 as *Aspergillus niger*. MALDI-TOF MS spectra for a few important bacterial strains (ADHLB-4: *Pseudomonas rhodesiae*; ADHLB-6: *Pseudomonas tolaasii*; ADHLB-3: *Pseudomonas fluorescens*; and ADHLB-7: *Janthinobacterium lividum*) indicating the protein profiling (2–20 KDa) are given in Fig. 3. A total of 10 microbial species (5 bacteria, 3 actinomycetes and 2 fungi) at site 1; 17 microbial species (8 bacteria, 4 actinomycetes and 5 fungi) at site 2; 10 microbial species (5 bacteria, 3 actinomycetes and 2 fungi) at site 3; and 8 microbial species (4 bacteria, 2 actinomycetes and 2 fungi) at site 4 were successfully isolated and identified. Maximum microbial diversity was recorded at site 2 whereas, minimum microbial diversity was recorded at site 4. The identification report was made using the EzBioCloud Database maintained at the facilitation center (Yoon et al. 2017). All microbial strains perceived using the 16S rRNA/18S rRNA gene sequencing are presented in Table 4 along with their accession number (received after submitting the sequences to the GenBank, NCBI in Fasta format) and level of closeness to its closest neighbor.

**Table 3:** Microbial diversity dwelling the high altitude snow-fed lake Hemkund.

S. No.	Microbial Diversity	Strain ID	Site 1	Site 2	Site 3	Site 4
**A**	**Bacteria**					
1	*Ralstonia eutropha*	ADHLB-4	+	+	-	+
2	*Neomicrococcus aestuarii*	ADHLB-2	-	+	-	+
3	*Pseudomonas rhodesiae*	ADHLA-8	+	+	+	-
4	*Pseudomonas fluorescens*	ADHLB-3	-	+	+	-
5	*Pseudomonas extremorientalis*	ADHLB-5	+	+	-	-
6	*Janthinobacterium lividum*	ADHLB-7	+	-	-	+
7	*Staphylococcus aureus*	ADHLB-8		+	+	-
8	*Bacillus licheniformis*	ADHLA-4	+	-		+
9	*Paenibacillus glucanolyticus*	ADHLB-1	-	+	+	-
10	*Pseudomonas tolaasii*	ADHLB-6	-	+	+	-
**B.**	**Actinomycetes**					
11	*Streptomyces clavifer*	ADHLA-2	-	+	+	+
12	*Streptomyces rangoon*	ADHLA-5	+	+	+	-
13	*Microbacterium schleiferi*	ADHLA-6	+	+	+	+
14	*Arthrobacter polychromogenes*	ADHLA-7	+	+	-	-
**C**	**Fungi**					
15	*Aspergillus cvjetkovicii*	ADHLF-1	+	+	-	-
16	*Aspergillus sydowii*	ADHLF-2	-	+	+	+
17	*Cladosporium fulvum*	ADHLF-7	-	+	+	+
18	*Aspergillus jensenii*	ADHLF-3	+	+	-	-
19	*Aspergillus niger*	ADHLF-5		+	-	-
**Total Microbes**			**10**	**17**	**10**	**08**

Abbreviation: +: Present; -: Absent

**Table 4:** Sequencing analysis of microbes isolated from water samples.

S. No.	Name of microbe	Strains	Nucleotide length (bp)	Accession numbers	Identity (%)	Database
1.	*Aspergillus cvjetkovicii*	ADHLF-1	542	OK576943	98.70	NCBI
2.	*Aspergillus sydowii*	ADHLF-2	371	OK576944	99.73	NCBI
3.	*Aspergillus jensenii*	ADHLF-3	543	OK576940	98.34	NCBI
4.	*Aspergillus cvjetkovicii*	ADHLF-7	554	OK576942	98.89	NCBI
5.	*Bacillus licheniformis*	ADHLA-4	1365	OK617288	99.85	NCBI
6.	*Paenibacillus glucanolyticus*	ADHLB-1	1377	OK617301	99.78	NCBI
7.	*Neomicrococcus aestuarii*	ADHLB-2	1158	OK617303	99.91	NCBI

**Fig. 4:**
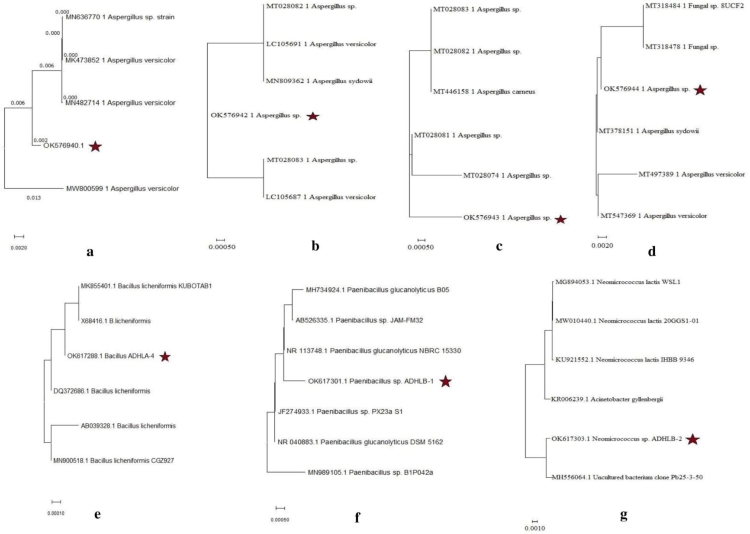
Phylogenetic trees; a: OK576940 (*Aspergillus* sp.); b: OK576942 (*Aspergillus* sp.); c: OK576943 (*Aspergillus* sp.); d: OK576944 (*Aspergillus* sp.); e: OK617288 (*Bacillus* sp.); f: OK617301 (*Paenibacillus* sp.); g: OK617303 (*Neomicrococcus* sp.).

The submitted sequences in GenBank, NCBI got the accession numbers (OK576940 for strain ADHLF-3; OK576942 for strain ADHLF-7; OK576943 for strain ADHLF-1; OK576944 for strain ADHLF-2; OK617288 for strain ADHLA-4; OK617301 for strain ADHLB-1; and OK617303 for strain ADHLB-2). Then using MEGA software it generated the optimal trees with the sum of branch length written just below the constructed tree for each strain as shown in Fig. 4 and percentage of replicate trees in which the associated taxa clustered together in the bootstrap test presented next to the branches. The evolutionary distances were computed using the maximum composite likelihood method and are in the units of the number of base substitutions per site (Mohapatra et al. 2014). Psychrophilic bacteria incorporate Gram-negative genera like Psychrobacter, Flavobacterium, Polaromonas, Polaribacter, and Pseudomonas; Gram-positive genera like Arthrobacter, Bacillus, Micrococcus species; microalgae, for example, Chlamydomonadales and a few types of archaea, yeasts, and fungi. *Pseudomonas fluorescens* is responsible for the production of Alanine racemase enzymes that has been used as antibacterial agent and in food storage (Yokoigawa et al., 2001).

## 4 Discussion

Air and water temperatures are closely associated with each other. Fluctuation in any of the temperatures must fluctuate the other temperature. The low temperature of water and air is because of cold winds, snow and ice that is the source of water and location of the lake at high altitude. Similar observations were also reported by Sharma and Kumar (2017) for Satopanth Lake, Kumar et al. (2019) for Dodi Tal, Kumar and Sharma (2019) for Neel Tal, and Deep et al. (2020) for Hemkund Lake. These previously mentioned studies recorded an air temperature between 3.5°C to 5.8°C and water temperature between 3.9°C to 7.6°C. In the current study, the pH value for water samples of Hemkund Lake was reported as slightly alkaline during the three years of the study period. pH is a logarithmic scale used to indicate or classify the acidity or alkalinity of water Sharma and Kumar (2017). & Kumar and Sharma (2019) recorded it between 6.85 to 7.10 for Satopanth Lake, and between 6.8 to 7.3 for Neel Tal located in the Garhwal Himalaya. TDS value describes the aggregate sum of portable charged particles, including minerals, salts or metals disintegrated in a given volume of water. TDS is the total of inorganic and organic matter. TDS values were recorded highest at site 2 and lowest at all other sites. It was because of human interference at site 2. A large number of pilgrims visited this place and took holy baths in this lake. A low range of TDS (72 to 79 mg.l^–1^) was recorded by Kumar and Sharma (2019) for Neel Tal; Kumar et al. (2019) recorded it between 22.5 to 241 mg.l^–1^ for Dodi Tal.

In simple terms, we can define turbidity as the measurement of clarity of the water. Its measurement is an important test to evaluate water quality. An aquatic body's bottom visibility is inversely proportional to the turbidity of water, as the turbidity enhances it reduces the bottom visibility. It was minimum (0.37 NTU) at site 4 and maximum (1.89 NTU) at site 2. It was because of snow melting at higher elevations and heavy rains that carried a lot of soil and debris along with water and dumped it into the lake Kumar et al. (2019). recorded it between 0.04 NTU to 1.79 NTU for Dodi Tal. The amount of DO in an aquatic body is inversely proportional to water temperature. High water temperature reduces the amount of dissolved oxygen and vice-versa (Rana et al. 2018). In the current study, the highest value of DO was recorded at site 1 whereas, the lowest value was recorded at site 2. This lowest value was because of high water temperature, air temperature and high pollution levels. Similar observations were also recorded by Deep et al. (2020) for Hemkund Lake (6.4 to 7.8 mg.l^−1^); Kumar and Sharma (2019) for Neel Tal (6.0 mg.l^−1^ to 6.6 mg.l^−1^); Kumar et al. (2019) for Dodi Tal (8.9 to 12.6 mg.l^−1^); Sharma and Kumar (2017) for Satopanth Lake (5.8 to 6.0 mg.l^−1^); and Rawat et al. (2021) for Tarakund Lake (5.6 to 8.6 mg.l^−1^). BOD of water samples was ranged from 0.2 to 1.2 mg.l^–1^. It was found maximum at sites 2 and 4 whereas, minimum at site 1 Deep et al. (2020). reported BOD for Hemkund Lake (0.8 to 2.6 mg.l^−1^); Kumar et al. (2019) for Dodi Tal (0.2 to 1.1 mg.l^−1^); and Rawat et al. (2021) for Tarakund Lake (0.4 to 2.2 mg.l^−1^).

Free CO_2_ has been considered as one of the most important parameters to find out the health status and water quality of a freshwater body. High free CO_2_ concentration represents the higher pollution level in a water body. The minimum value (2.2 mg.l^–1^) and maximum value (11.0 mg.l^–1^) were recorded during the study period Deep et al. (2020). reported free CO_2_ for Hemkund Lake (2.2 to 6.6 mg.l^−1^); Kumar and Sharma (2019) for Neel Tal (4.4 to 8.8 mg.l^−1^); Kumar et al. (2019) for Dodi Tal (2.2 to 4.84 mg.l^−1^); Rawat et al. (2021) for Tarakund Lake (2.2 to 11.0 mg.l^−1^). Total hardness can be expressed in the form of CaCO_3_. Total hardness was reported from 4 to 24 mg.l^–1^ during the study period. Various observations for total hardness were recorded by Deep et al. (2020) for Hemkund Lake (6 to 12 mg.l^−1^); Kumar and Sharma (2019) for Neel Tal (20 to 24 mg.l^−1^); Kumar et al. (2019) for Dodi Tal (15.8 to 38.8 mg.l^−1^); Sharma and Kumar (2017) for Satopanth Lake (22 to 22.5 mg.l^−1^); and Rawat et al. (2021) for Tarakund Lake (24 to 62 mg.l^−1^). As water travels through soil and rock, it dissolves tiny measures of minerals and holds them in arrangement (Oram 2018). The concentrations of calcium and magnesium were recorded within a range of 1.49 to 4.74 mg.l^–1^ and 0.61 to 1.63 mg.l^–1^. Similar kinds of findings were also recorded by Deep et al. (2020) for Hemkund Lake (0.8 to 1.6 mg.l^−1^ for calcium and 0.98 to 1.47 mg.l^−1^ for magnesium); Kumar and Sharma (2019) for Neel Tal (5.6 to 6.4 mg.l^−1^ for calcium and 1.47 to 2.44 mg.l^−1^ for magnesium); Sharma and Kumar (2017) for Satopanth Lake (7.89 to 7.95 mg.l^−1^ for calcium and 0.53 to 0.66 mg.l^−1^ for magnesium) and Torres and Monge (1998) for Mexican lake (1.77 mg.l^−1^ for calcium and 4.80 mg.l^−1^ for magnesium).

Total alkalinity is an estimation of the water's capacity to oppose the change in pH. Maximum alkalinity was reported at sites 3 and 4 whereas, the lowest was at site 2. It was ranged between 8 to 50 mg.l^−1^. Moderately high alkalinity (70 mg.l^–1^) was also observed by Torres and Monge (1998) for Mexican lake. EC reflects the water's tendency to conduct electrical current and has a direct relationship with water's dissolved ion concentration. It was ranged between 27.93 to 384.5 mg.l^−1^. Similar observations were recorded by Deep et al. (2020) for Hemkund Lake (144 µS/cm to 171µS/cm); Kumar and Sharma (2019) for Neel Tal (135 µS/cm to 142µS/cm); Kumar et al. (2019) for Dodi Tal (42.5 µS/cm to 449 µS/cm) and the maximum was recorded by Ghimire et al. (2013) for Sagarmatha lakes in Nepal (0.021 mS/cm to 0.30 mS/cm). Chloride was reported between 118 to 12.32 mg.l^−1^. Various concentration were recorded by Deep et al. (2020) for Hemkund Lake (7.10 to 11.36 mg.l^−1^); Kumar and Sharma (2019) for Neel Tal (11.36 mg.l^−1^); Kumar et al. (2019) for Dodi Tal (4.26 to 14.2 mg.l^−1^) and Sharma and Kumar (2017) for Satopanth Lake (9.14 to 9.90 mg.l^−1^). Sulphates usually occur as a principal ion in water. The maximum value for sulphates was recorded to be 0.171 mg.l^−1^ during the study period. Similar kind of data was reported by Deep et al. (2020) for Hemkund Lake (0.203 to 0.287 mg.l^−1^); Kumar and Sharma (2019) for Neel Tal (0.307 to 0.342 mg.l^−1^) and Sharma and Kumar (2017) for Satopanth Lake (0.730 mg.l^−1^).

Phosphates concentration was recorded within a range of 0.48 to 0.191 mg.l^−1^ whereas, nitrates concentration was ranged between 0.100 to 0.574 mg.l^−1^. Many studies were recorded similar observations. These include the work of Deep et al. (2020) for Hemkund Lake (0.067 to 0.084 mg.l^−1^ for phosphates and 0.361 to 0.513 mg.l^−1^ for nitrates); Kumar and Sharma (2019) for Neel Tal (0.064 to 0.079 mg.l^−1^ for phosphates and 0.631 to 0.713 mg.l^−1^ for nitrates); Kumar et al. (2019) for Dodi Tal (0.015 to 0.180 mg.l^−1^ for phosphates and 0.018 to 0.220 mg.l^−1^ for nitrates); Sharma and Kumar (2017) for Satopanth Lake (0.060 to 0.062 mg.l^−1^ for phosphates and 0.130 mg.l^−1^ for nitrates) and Torres and Monge (1998) for Mexican lake (0.185 to 0.574 mg.l^−1^ for phosphates and 0.675 to 1.050 mg.l^−1^ for nitrates). Fecal coliforms are facultative anaerobic, rod-shaped, gram-negative, non-sporulating bacteria. No fecal coliform was reported at any of the sampling sites during the study period. This has confirmed that there was no contamination of human or fecal waste. Total coliforms were recorded within a range of 28 CFU/100 ml to 1321 CFU/100 ml. Maximum total coliforms were reported at site 2 whereas, minimum total coliforms were reported at site 4. The maximum total coliforms at site 2 are because of the devotees as they took bath at the same site. Site 2 was the most disturbing site among all the sampling sites. Total coliform incorporates microscopic organisms that are found in the soil, in surface water contaminated with excreta. Fecal coliforms are the group of the total coliforms that are considered to be present explicitly in the gut and feces of warm-blooded creatures. Similar observations were observed by Deep et al. (2020) for Hemkund Lake, Kumar and Sharma (2019) for Neel Tal, Kumar et al. (2019) for Dodi Tal, and Rawat et al. (2021) for Tarakund Lake.

Most of the microbes isolated and recognized were gram-positive. These bacteria are stress-resistant and long-range migrants, particularly the actinobacteria (Cerritos et al. 2011; Kumar et al. 2014). During the research period, an aggregate of 19 microbial species were identified that included 10 bacterial species, 4 actinomycetes species and 5 fungal species. Although, the microbial diversity was evenly distributed in the lake water but *Microbacterium schleiferi* is the only species that was found at all the sampling sites. Four species of Pseudomonas were identified in the water samples. Three out of these four species (*Pseudomonas rhodesiae*, Pseudomonas fluorescens and *Pseudomonas tolaasii*) are the members of *Pseudomonas fluorescens* group. A similar kind of microbial diversity was also recorded by Kumar and Sharma (2020c) for Satopanth Lake and Neel Tal, Kumar and Sharma (2020d) for Dodi Tal. Nutrient factors, hydrological conditions, and other environmental parameters including the dissolved oxygen, pH, turbidity, and nutrient concentrations play roles in determining or shaping the microbial diversity in lakes, rivers, and reservoirs (Wu et al., 2006; Zhong et al., 2016; Bull et al., 2016; Núñez Salazar et al. 2020; Wang et al. 2021).

## 5 Importance of psychrophiles

Cold-loving microbes can be used for the production of extremozymes. These extremozymes have commercial and economic values hence can be used in industries at a large scale and also used for bioremediation of contaminated soils and wastewaters (Margesin and Feller, 2010). Psychrophiles primarily act as environmental cleaners. They can remove hydrocarbon pollutants in cryo conditions (Morita, 1975). These can be used in the food and beverage industries (Feller and Gerday, 2003). These can be used to produce lactose-free milk for lactose intolerant people (Silanikove et al., 2015). Enzymes isolated from the psychrophiles can be used in detergent formulations like proteases, lipases, and α-enzymes (Hasan et al., 2010). *Pseudomonas fluorescens* group of bacteria can be used as important biocontrol agents to suppress plant diseases and fungal infection (Hoffland et al. 1996, Wei et al. 1996). Psychrophilic microorganisms play crucial roles in biogeochemical cycles (Junge et al., 2019). Far off high mountain lakes, being a long way from residence and situated in outrageous conditions, get less effect from human exercises but magnify the impacts of worldwide environment changes, and can accordingly be taken as a reflection of regular ecological changes (Patrick, 1998).

## 6 Conclusion

The current study discusses the various environmental and physicochemical factors that influence the microbial diversity at various sampling sites of high altitude snow-fed lake Hemkund located in India. No fecal coliform was reported during the study period. It ensures that there is no contamination of any kind of fecal waste in Hemkund Lake. Dissolved oxygen concentration also represents the health status of Hemkund Lake at each sampling site. pH is slightly alkaline or just near to neutral that may be considered as a hope for better water quality and health status. Although there is less microbial diversity available in water few of the microbial species are very toxic and severely affect the water quality. Most of the identified bacteria were gram-positive. The α-diversity of this lake was 19 including 10 species of bacteria, 4 species of actinomycetes and 5 species of fungi during the study period. Three *Pseudomonas* species (*Pseudomonas rhodesiae, Pseudomonas fluorescens* and *Pseudomonas tolaasii*) are belongs to *Pseudomonas fluorescens* group. Psychrophilic microbial diversity is of paramount importance as they act as environmental cleaners and also play a key role in various biogeochemical cycles that are very much important for life on Earth. The current study will provide baseline data to researchers working in a similar discipline. There is also a bright possibility of identifying novel species of microorganisms in this lake because of its location and harsh-environmental conditions.

## CRediT authorship contribution statement

**Vidhu Gupta:** Supervision, Writing – review & editing. **Somashekar Chandran:** Writing – review & editing. **Akash Deep:** Data curation, Writing – review & editing. **Rahul Kumar:** Conceptualization, Writing – original draft, Writing – review & editing. **Lalita Bisht:** Writing – review & editing.

## Declaration of Competing Interest

The authors declare that they have no known competing financial interests or personal relationships that could have appeared to influence the work reported in this paper.

## References

[bib0001] APHA (2012).

[bib0002] Bull A.T., Asenjo J.A., Goodfellow M., Gomez-Silva B. (2016). The Atacama desert: technical resources and the growing importance of novel microbial diversity. Annu. Rev. Microbiol..

[bib0003] Catalan G., Janssens A., Rispens G., Csiszar S., Seeck O., Rijnders G., Noheda B. (2006). Polar domains in lead titanate films under tensilestrain. Phys. Rev. Lett..

[bib0004] Cerritos R., Eguiarte L.E., Avitia M., Siefert J., Travisano M. (2011). Diversity of culturable thermo-resistant aquatic bacteria along an environmental gradient in Cuatro Cienegas, Mexico. Antonie Leeuwenhoek..

[bib0005] Clesceri L.S., Greenberg A.E., Eaton A.D. (1998).

[bib0006] De Maayer P., Anderson D., Cary C., Cowan D.A. (2014). Some like it cold: understanding the survival strategies of psychrophiles. EMBO Rep..

[bib0007] Deep A., Gupta V., Bisht L., Kumar R. (2020). Application of WQI for water quality assessment of high-altitude snow-fed sacred Lake Hemkund, Garhwal Himalaya. Sustain. Water Resour. Manag..

[bib0008] Dong H., Jiang H., Yu B., Liu X., Zhang C. (2010). Impacts ofenvironmental change and human activity on microbial ecosystems onthe Tibetan Plateau, NW China. GSA Today.

[bib0009] Feller G., Gerday C. (2003). Psychrophilic enzymes: hot topics in cold adaptation. Nat. Rev. Microbiol..

[bib0010] Fenselau C., Demirev P.A. (2001). Characterization of intact microorganisms by MALDI mass spectrometry. Mass Spectrom. Rev..

[bib0011] Ghimire N.P., Jha P.K., Caravello G. (2013). Water quality of high-altitude lakes in Sagarmatha (Everest) National Park, Nepal. J. Environ. Prot..

[bib0012] Hammer Ø., Harper D.A.T., Ryan P.D. (2001). PAST: palentological statistics software package for education and data analysis. Palaeontol. Electron..

[bib0013] Hasan F., Shah A.A., Javed S., Hameed A. (2010). Enzymes used in detergents: Lipases. Afr. J. Biotechnol..

[bib0014] Hoffland E., Halilinen J., Van Pelt J.A. (1996). Comparison of systemic resistance induced by avirulant and nonpathogenic Pseudomonas species. Phytopathology.

[bib0015] Junge, K., Cameron, K., Nunn, B. 2019. Diversity of Psychrophilic bacteria in sea and glacier ice environments—insights through genomics, metagenomics, and proteomics approaches. In. Microbial Diversity in the Genomic Era. pp. 197–216. 10.1016/B978-0-12-814849-5.00012-5.

[bib0016] Kumar M., Yadav A.N., Tiwari R., Prasanna R., Saxena A.K. (2014). Evaluating the diversity of culturable thermotolerant bacteria from four hot springs of India. J. Biodivers. Bioprospect. Dev..

[bib0017] Kumar R., Singh S., Sharma R.C. (2018). Application of WQI for assessment of water quality of high altitude lake Dodi Tal, Garhwal Himalaya, India. Sustain. Water Resour. Manag..

[bib0018] Kumar R., Sharma R.C. (2019). Assessment of the water quality of Glacier-fed lake Neel Tal of Garhwal Himalaya, India. Water Sci..

[bib0019] Kumar R., Sharma R.C. (2020). Thermophilic microbial diversity and physicochemical attributes of thermal springs in the Garhwal Himalaya. Environmental and Experimental Biology.

[bib0020] Kumar R., Sharma R.C. (2020). Microbial diversity in relation to physico-chemical properties of hot water ponds located in the Yamunotri landscape of Garhwal Himalaya. Heliyon.

[bib0021] Kumar R., Sharma R.C. (2020). Exploration of Psychrophilic Microbial Diversity and Physicochemical Environmental Variables of Glacier-Fed Lakes in the Garhwal Himalaya, India. Taiwan Water Conserv..

[bib0022] Kumar R., Sharma R.C. (2020). Assessment of Water Quality Parameters and Identification of Microbial Diversity in the High Altitude Sacred Lake Dodi Tal, Garhwal Himalaya. Taiwan Water Conserv.

[bib0023] Kumar R., Sharma R.C. (2021). Psychrophilic microbial diversity and physicochemical characteristics of glaciers in the Garhwal Himalaya, India. J. Microbiol., Biotechnol. Food Sci..

[bib0024] Ley R.E., Bäckhed F., Turnbaugh P., Lozupone C.A., Knight R.D., Gordon J.I. (2005). Obesity alters gut microbial ecology. Proc. Natl. Acad. Sci.

[bib0025] Liao B., Yan X., Zhang J., Chen M., Li Y., Huang J., Wang J. (2019). Microbial community composition in alpine lake sediments from the Hengduan Mountains. Microbiol. Open.

[bib0026] Liu L., Yang J., Yu Z., Wilkinson D.M. (2015). The biogeography ofabundant and rare bacterioplankton in the lakes and reservoirs of China. ISME J..

[bib0027] Liu C., Yao M., Stegen J.C., Rui J., Li J., Li X. (2017). Long-term nitrogenaddition affects the phylogenetic turnover of soil microbial community responding to SWC pulse. Sci. Rep..

[bib0028] Margesin R., Feller G. (2010). Biotechnological applications of psychrophiles. Environ. Technol..

[bib0029] Merino N., Aronson H.S., Bojanova D.P., Feyhl-Buska J., Wong M.L., Zhang S., Giovannelli D. (2019). Living at the extremes: extremophiles and the limits of life in a planetary context. Front. Microbiol..

[bib0030] Mohapatra S., Samantaray D.P., Samantaray S.M. (2014). Phylogenetic heterogeneity of the rhizospheric soil bacterial isolates producing PHAs revealed by comparative analysis of 16s-rRNA. Int. J. Curr. Microbiol. App. Sci..

[bib0031] Mohapatra S., Samantaray D.P., Samantaray S.M., Mishra B.B., Das S., Majumdar S., Pradhan S.K., Rath S.N., Rath C.C., Akthar J., Achary K.G. (2016). Structural and thermal characterization of PHAs produced by Lysinibacillus sp. through submerged fermentation process. Int. J. Biol. Macromol..

[bib0032] Mohapatra S., Pattnaik S., Maity S., Mohapatra S., Sharma S., Akhtar J., Pati S., Samantaray D.P., Varma A. (2020). Comparative analysis of PHAs production by Bacillus megaterium OUAT 016 under submerged and solid-state fermentation. Saudi J. Biol. Sci..

[bib0033] Morita R. (1975). Psychrophilic bacteria. Bacteriol. Rev..

[bib0034] Nazir, R., Rehman, S., Nisa, M., Ali Baba, U. 2019. Exploring bacterial diversity: from cell to sequence. In Freshwater Microbiology pp. 263–306.

[bib0035] Newton R.J., Jones S.E., Eiler A., McMahon K.D., Bertilsson S. (2011). A guide to the natural history of freshwater lake bacteria. Microbiol. Mol. Biol. Rev..

[bib0036] Núñez Salazar R., Aguirre C., Soto J., Salinas P., Salinas C., Prieto H., Paneque M. (2020). Physicochemical parameters affecting the distribution and diversity of the water column microbial community in the high-altitude Andean lake system of La Brava and La Punta. Microorganisms.

[bib0037] Oarga A. (2009). Life in extreme environments. J. Biol. Earth Sci..

[bib0038] Oram, B. 2018. Hard water hardness calcium magnesium water corrosion mineral scale. Water Res. Center. https://water-research.net/index.php/water-treatment/tools/hard-water-hardness.

[bib0039] Patrick S. Battarbee R.W. Wathne B. Psenner R. 1998. Measuring and modelling the dynamic response of remote mountain lake ecosystems to environmental change: an introduction to the MOLAR project. Hydrology, Water Resources and Ecology in Headwaters Proceedings of the HeadWater'98 Conference (Kovar UTK Peters N.E. Craig R.G., eds), pp. 403–410.

[bib0040] Pandit M.K., Manish K., Koh L.P. (2014). Dancing on the roof of the world: ecological transformation of the Himalayan landscape. BioScience.

[bib0042] Pérez M.T., Sommaruga R. (2006). Differential effect of algal- and soil-derived dissolved organic matter on alpine lake bacterial community composition and activity. Limnol. Oceanogr..

[bib0043] Pryce T.M., Palladino S., Kay I.D., Coombs G.W. (2003). Rapid identification of fungi by sequencing the ITS l and ITS2 regions using an automated capillary electrophoresis system. Med. Mycol..

[bib0044] Rahi P., Sharma O.P., Shouche Y.S. (2016). Matrix-Assisted Laser Desorption/Ionization Time-of-Flight Mass-Spectrometry (MALDI-TOF MS) based microbial identifications: challenges and scopes for microbial ecologists. Front. Microbial..

[bib0045] Raja H.A., Miller A.N., Pearce C.J., Oberlies N.H. (2017). Fungal identification using molecular tools: a primer for the natural products research community. J. Nat. Prod..

[bib0046] Rampelotto P.H. (2010). Resistance of microorganisms to extreme environmental conditions and its contribution to astrobiology. Sustainability.

[bib0047] Rana K.S., Sharma R.C., Tivari V., Kumar R. (2018). Assessment of surface water quality of the Himalayan lake Beni Tal, India. Curr. Res. Hydrol. Water Resour..

[bib0048] Rawat H., Singh R., Namtak S., Deep A., Mamgain S., Sharma A., Tripathi N., Kirti V., Kumar R. (2021). Water quality assessment of Garhwal Himalayan Lake Tarakund based on the application of WQI and mitigation measures for its conservation and management. Int. J. Energ. Water Res..

[bib0049] Rohomania T., Saha M.L., Hossain A., Rahman M.S. (2015). Morphological and biochemical characterization of bacteria isolated from fresh and salted hilsa, Tenualosa ilisha (Hamilton, 1822). Bangladesh J. Microbiol..

[bib0050] Rose K.C., Williamson C.E., Saros J.E., Sommaruga R., Fischer J.M. (2009). Differences in uv transparency and thermal structurebetween alpine and subalpine lakes: Implications for organisms. Photochem. Photobiol. Sci..

[bib0051] Saitou N., Nei M. (1987). The neighbor-joining method- a new method for reconstructing phylogenetic trees. Mol. Biol. Evol..

[bib0052] Sharma B., Verma R., Dev K., Thakur R. (2012). Molecular characterization of Manikaran hot spring microbial community by 16S rRNA and RAPD analysis. BioTechnol. Indian J..

[bib0053] Sharma R.C., Kumar R. (2017). Water quality assessment of sacred glacial Lake Satopanth of Garhwal Himalaya, India. Appl. Water Sci..

[bib0054] Silanikove N., Leitner G., Merin U. (2015). The interrelationships between lactose intolerance and the modern dairy industry: global perspectives in evolutional and historical backgrounds. Nutrients.

[bib0056] Takahashi S., Tomita J., Nishioka K., Hisada T., Nishijima M. (2014). Development of a prokaryotic universal primer for simultaneous analysis of bacteria and archaea using next-generation sequencing. PLOS ONE.

[bib0057] Tamura K., Stecher G., Peterson D., Filipski A., Kumar S. (2013). MEGA 6: molecular evolutionary genetics analysis version 6.0. Mol. Biol. Evol..

[bib0058] Thompson J.D., Higgins D.G., Gibson T.J. (1994). CLUSTAL W: improving the sensitivity of progressive multiple sequence alignment through sequence weighting, position-specific gap penalties and weight matrix choice. Nucl. Acids Res..

[bib0059] Torres C.A., Monge R.C. (1998). Water quality characteristics of a high altitude oligotrophic Mexican lake. Aquat. Ecosyst. Health Manag..

[bib0060] Wang J., Fan H., He X., Zhang F., Xiao J., Yan Z., Feng J., Li R. (2021). Response of bacterial communities to variation in water quality and physicochemical conditions in a river-reservoir system. Glob. Ecol. Conserv..

[bib0061] Wasserstrom H., Kublik S., Wasserstrom R., Schulz S., Schloter M., Steinberger Y. (2017). Bacterial community composition in costaldunes of the Mediterranean along a gradient from the sea shore to the inland. Sci. Rep..

[bib0062] Wei G., Kloepper J.W., Tuzun S. (1996). Induced systemic resistance to cucumber diseases and increased plant growth by plant growth promoting rhizobacteria under field condition. Phytopathology.

[bib0063] Wu Q.L., Zwart G., Schauer M., Kamst-Van Agterveld M.P., Hahn M.W. (2006). Bacterioplankton community composition along a salinity gradient of sixteen high-mountain lakes located on the Tibetan Plateau, China. Appl. Environ. Microbiol..

[bib0064] Yang J., Jiang H., Dong H., Liu Y. (2019). A comprehensive census of lake microbial diversity on a global scale. Sci. China.

[bib0065] Yokoigawa K., Okubo Y., Kawai H., Esaki N., Soda K. (2001). Structure and function of Psychrophilic Alanine racemase. J. Mol. Catal. B: Enzymatic.

[bib0066] Yoon S.H., Ha S.M., Kwon S., Lim J., Kim Y., Seo H., Chun J. (2017). Introducing EzBioCloud: a taxonomically united database of 16S rRNA gene sequences and whole-genome assemblies. Int. J. Syst. Evol. Microbiol..

[bib0067] Zhong Z.-P., Liu Y., Miao L.-L., Wang F., Chu L.-M., Wang J.-L., Liu Z.-P. (2016). Prokaryotic community structure driven by salinity and ionic concentrations in plateau lakes of the Tibetan plateau. Appl. Environ. Microbiol..

